# Identifying right and left impact using the derivative of linear resultant acceleration from a single sacrum-mounted IMU

**DOI:** 10.1017/wtc.2025.4

**Published:** 2025-02-28

**Authors:** Aida Chebbi, Rachel M. Robinson, Seth R. Donahue, Michael E. Hahn

**Affiliations:** 1 Knight Campus for Accelerating Scientific Impact, University of Oregon, Eugene, OR, USA; 2 Department of Human Physiology, University of Oregon, Eugene, OR, USA; 3 Shriners Children’s Hospital, Lexington, KY, USA

**Keywords:** angular velocity, crackle, inertial measurement units, initial contact, running monitoring, toe-off

## Abstract

This study introduces a novel method for gait analysis using a single inertial measurement unit placed on the sacrum. This method is valid not only on level ground but also on incline and decline conditions. The method leverages the “crackle” function, the third derivative of the sacral resultant acceleration, to identify right and left gait events. This approach is particularly effective in capturing the initial peak in acceleration data during foot impact with the ground, often overlooked by other methods. The study aimed to demonstrate the method’s accuracy in identifying the right- and left-side impacts during level ground, incline, and decline runs across a range of speeds. Additionally, the algorithm was applied in outdoor running scenarios, where it performed very well, further validating its robustness and reliability. The results are compared with other existing methods to highlight the effectiveness of this approach.

## 1. Introduction

Gait analysis, a thorough examination of human movement, is fundamental in various fields such as sports science, rehabilitation, and clinical diagnostics (Yang et al., 2024). Traditional methods often require complex setups with multiple sensors, cameras, force plates, and instrumented treadmills, which can affect natural movement patterns. However, the introduction of wearable sensors, especially inertial measurement units (IMUs), has transformed gait analysis (Liu et al., 2005). These sensors provide a cost-effective and practical alternative to traditional three-dimensional motion capture systems while still offering comparable accuracy in monitoring running kinetics and kinematics.

**Figure 1.**
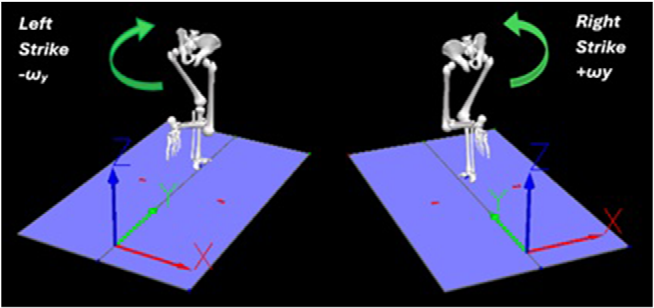
Pelvis motion associated with right initial contact (RIC; positive angular velocity about the anterior-posterior axis +ω_y_) and left initial contact (LIC; negative angular velocity about the anterior-posterior axis −ω_y_) (Wixted et al., 2010).

In a study conducted by Wada et al. (2020) on pelvic orientation during sprinting, a single inertial sensor was used to measure the pelvic orientation angles. The sensor was mounted on the lower back of each sprinter, and the data collected provided valuable insights into the changes in pelvic orientation during different phases of sprinting. This suggests that pelvic orientation can vary significantly between the right and left leg support phases during sprinting (Figure 1).

To assess the IMU accuracy compared to a motion capture system (MOCAP), the root mean squared error (RMSE), and Pearson’s correlation coefficient between IMU and MOCAP were computed for each trial. The absolute angle RMSE between the IMU and MOCAP was 4.1° for roll, 2.8° for pitch, and 3.6° for yaw for all trials. Pearson’s correlation coefficients were 0.88 for roll, 0.79 for pitch, and 0.97 for yaw, demonstrating strong linear relationships for all pelvic tilt angles. These values suggest that the IMU demonstrates sufficient accuracy and reliability for the outdoor assessment of pelvic tilt, making it a viable tool for such measurements according to Wada et al. (2020).

**Figure 2.**
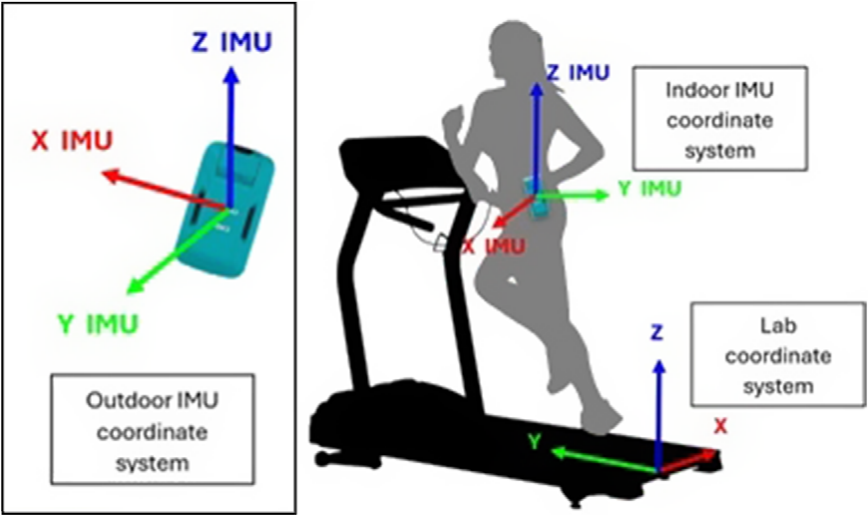
Laboratory and IMU coordinate systems.

**Figure 3.**
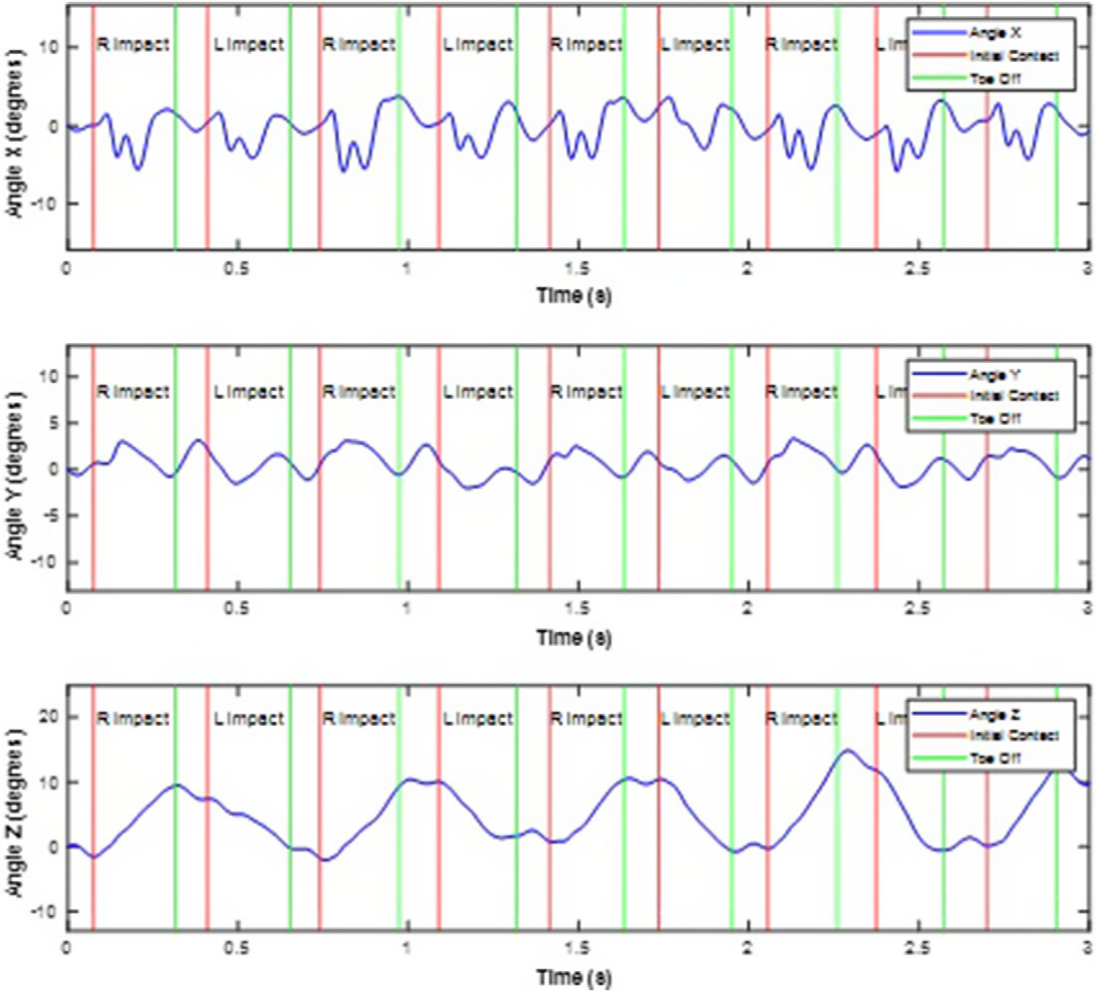
Representative time series of pelvic angular displacement integrated from angular velocity data collected by a sacral-mounted IMU.

The motion of the pelvis during sprinting can be described by examining the angular velocity about the anterior-posterior axis ω_y_ and the angle Y in degrees. Coordinate system axis definitions for the laboratory and sacral IMU are shown in Figure 2a and b. As the right foot approaches the ground, the pelvis begins to drop laterally, causing the angular velocity to increase. This velocity reaches its peak at the instance of right foot impact, indicating a rapid lateral drop of the pelvis. After the right foot makes contact with the ground, the pelvis starts to stabilize, and the angular velocity decreases as the lateral drop slows down. The pelvis then transitions to a more neutral position as the body shifts to the flight phase before left foot contact. This dynamic motion, characterized by changes in angular velocity, is crucial for efficient running, as it helps absorb impact forces and prepares the body for the next stride. Exemplar time series data for the sacral IMU are shown in Figure 3.

The use of a single IMU placed on the sacrum has proven to be highly effective in indicating whether the impact is right or left side during indoor running. Many studies have explored the approach of using one single IMU placed on the sacrum to detect right and left foot impact during controlled runs (Auvinet et al., 2002; Benson et al., 2019; Bergamini et al., 2012; Lee et al., 2010; Reenalda et al., 2021; Wixted et al., 2010). A recent study by Kiernan et al. (2023) reproduced these methods and evaluated their accuracy in identifying the side of foot impact. The study found that the Lee method (Lee et al., 2010) was the most accurate, correctly identifying impact side 81.9% of the time. However, these methods were only tested on level ground (LG) track and treadmill conditions. Accurate gait events (GEs) detection is crucial as it directly impacts the subsequent accuracy of side impact detection, ensuring the overall effectiveness of the algorithm. The performance of existing methods in detecting GEs provides context for this calculation. The Lee method (Lee et al., 2010) showed a bias of −20.3 ms for initial contact (IC) and −1.5 ms for terminal contact (TC). The Auvinet method (Auvinet et al., 2002) had a bias of −30.4 ms for IC and −2.8 ms for TC. The Benson method (Benson et al., 2019) demonstrated a bias of −25.6 ms for IC and −3.1 ms for TC. Despite these biases, the IMU demonstrated sufficient accuracy and reliability for detecting side impacts and GEs during running. This highlights the importance of precise GE detection as a foundational step that influences the accuracy of subsequent methods.

Therefore, there is a need for a more accurate method, especially for outdoor running scenarios, where the grade can differ from LG and the rhythm can be disrupted by unexpected events such as tripping, falling, or slowing down due to obstacles, changes in terrain, or fatigue. These incidents can alter the runner’s pace and stride, adding complexity to the cyclic nature of running. In response to this need, the recent study has been performed as part of a broader effort to identify right and left GEs using a single wearable device (IMU) placed on the sacrum not only on LG but also in incline and decline conditions.

The main goal is to detect the impact of the right and left legs, reducing the number of sensors traditionally needed for running assessment, thus enhancing user comfort and convenience of measurement in real-world applications, such as a marathon. Furthermore, this study compares the presented method with previously documented methods (Auvinet et al., 2002; Benson et al., 2019; Kiernan et al., 2023; Lee et al., 2010; Wixted et al., 2010).

The presented method is a significant step toward a more comprehensive and accurate method for gait analysis, particularly in outdoor running scenarios. The use of this method could lead to the development of targeted training programs to improve running performance and reduce the risk of injury. By using data from a single sacrum-mounted IMU, we can closely track important running metrics such as stride length, cadence, and ground reaction forces (Zeng et al., 2022). This provides real-time feedback, helping runners keep good form and balance. We can also identify small changes in pelvic tilt and rotation, which can warn of potential injuries early on. This method makes measurements more comfortable and convenient, giving runners personalized insights based on their unique running style, leading to safer and more effective training programs.

## 2. Materials and methods

### 2.1. Participants and protocol in the indoor setting

Ten healthy recreational runners, (7 F, 3 M, 25.5 ± 8.2 years, 168.6 ± 8.6 cm, 59.6 ± 7.1 kg) were equipped with multiaxis IMUs (Casio, Tokyo, JPN) on the dorsal aspect of the participants’ feet and approximately on the sacrum, clipped to the back of the participants’ waistband. The sensors recorded three-dimensional linear accelerations and angular velocities at a frequency of 200 Hz. The inertial data were postprocessed using a Kalman filter to align the vertical axis of the local (IMU) coordinate system with gravity.

Each participant ran on a force-instrumented treadmill (Bertec, Columbus, OH), which recorded data at 1000 Hz, at three different grades: LG, incline (IN), and decline (DE) at an angle of ±7.5°. The protocol included thirteen 30-s trials: five runs at LG; three paces slower than 5k race pace, one at 5k race pace, and one optional trial faster than 5k race pace. The same four initial runs at LG were then repeated at IN and DE. The total range of speeds was from 3.16 to 4.88 ms^-1^.

### 2.2. Participants and protocol in the outdoor setting

Seven healthy recreational runners (4 F, 3 M, 24.9 ± 6.0 years, 174.9 ± 15.1 cm, 65.6 ± 8.2 kg) as part of a larger data collection were equipped with the same IMUs (Casio, Tokyo, JPN) as in the indoor setting. Participants were asked to run a five-mile course near the University of Oregon and in surrounding parks. Participants also wore a Garmin GPS, (Kansas City, KS). The total range of speeds was from 3 to 5.5 ms^−1^. The IMU data were filtered with a fourth-order low-pass zero-lag Butterworth filter (fc = 35 Hz) and down-sampled to 100 Hz. Velocity and slope measured by the GPS from the participant were filtered with a zero-lag 10-s moving average filter. Velocities from GPS data were set to the nearest 0.25 ms^−1^ for speeds ranging from 3 to 5.5 ms^−1^, and all other speeds were removed from the analysis. Three different inclination measures were included from the measured GPS data; incline foot strikes were identified at measured slopes >5°, and decline foot strikes were identified at measured slopes <−5°. This was due to errors up to ±4° throughout the run. Velocity data were then matched to the beginning and end of the IMU and kinetic data.

### 2.3. Data processing

Several methods have been developed to identify right and left sides during LG running. Auvinet et al. (2002) proposed a method that identifies the stance side based on the mean value of the *z*-axis acceleration in a window encompassing the IC. If the magnitude is <0, it is classified as a left stance, and if it is more than 0, it is classified as a right stance.

Lee et al. (2010) developed a method that identifies the stance side by finding positive and negative peaks in *z*-axis acceleration between successive IC and TC events. The stance is labeled as left if the absolute value of the negative peak is greater, and as right if the absolute value of the positive peak is greater. Benson et al. (2019) proposed a method that identifies the stance side by finding the largest positive and negative peaks in the *z*-axis acceleration during each stance. The stance is set to the right when the largest positive peak in the *z*-axis acceleration is greater than the largest negative peak and is closer to the TC event, which is the moment when the foot is about to leave the ground.

**Figure 4.**
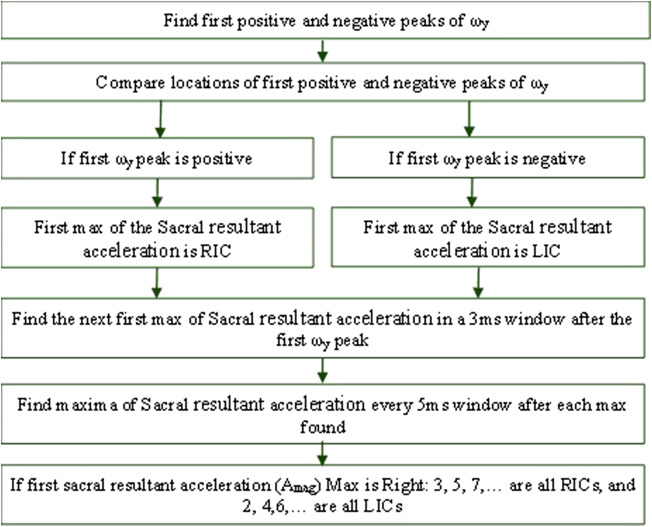
Flow chart for detecting right and left initial contact from the sacral IMU.

In addition, in a previous method developed by our group (Chebbi et al., 2023), the locations of the first maximum and the first minimum of the angular velocity about the anterior-posterior axis (ω_y_) were compared to determine whether the first extremum to consider is a minimum or a maximum under LG, IN, and DE conditions. Once this was established, the second extremum was identified within a 3-ms window. The interval between two successive minima/maxima was set at 5 ms. If the absolute value of the positive peak magnitude was greater than the absolute value of the negative peak magnitude, the first peak of the sacral resultant acceleration (Sacral A_mag_) after Sacral ω_y_ positive peak was identified as the right IC (RIC). However, if the absolute value of the negative peak height was greater, the first peak of sacral resultant acceleration after the Sacral ω_y_ negative peak was identified as the left IC (LIC) (Figure 4).

In the current work, we present a new approach that uses the third derivative of the acceleration, referred to as “crackle,” derived from the resultant acceleration (A_mag_) of the sacral IMU. If the acceleration function is represented as a polynomial *P*(*x*) Eq. (1) with coefficients 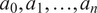 the “crackle” functions will be represented in Eq. (2)
(1)

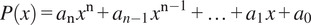


(2)

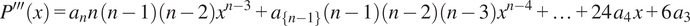



**Figure 5.**
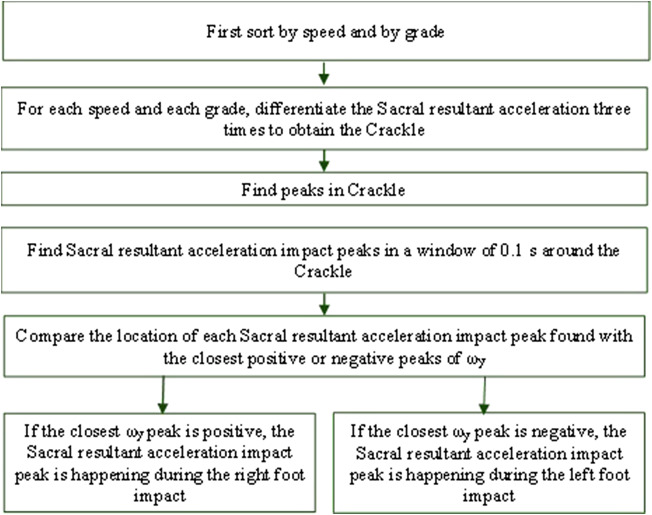
Flow chart of detecting right and left initial contact from the sacral IMU using both crackle and angular velocity about the anterior-posterior axis.

The identification process involves detecting the critical points where the “crackle” *P*”’(*x*) Eq. (2) becomes zero, indicating potential peaks or troughs. Within a 10-ms window around each A_mag_ peak (Figure 5), points where *P*”’(*x*) = 0 are detected. These critical points correspond to the moments of extremum in the “crackle” function.

**Figure 6.**
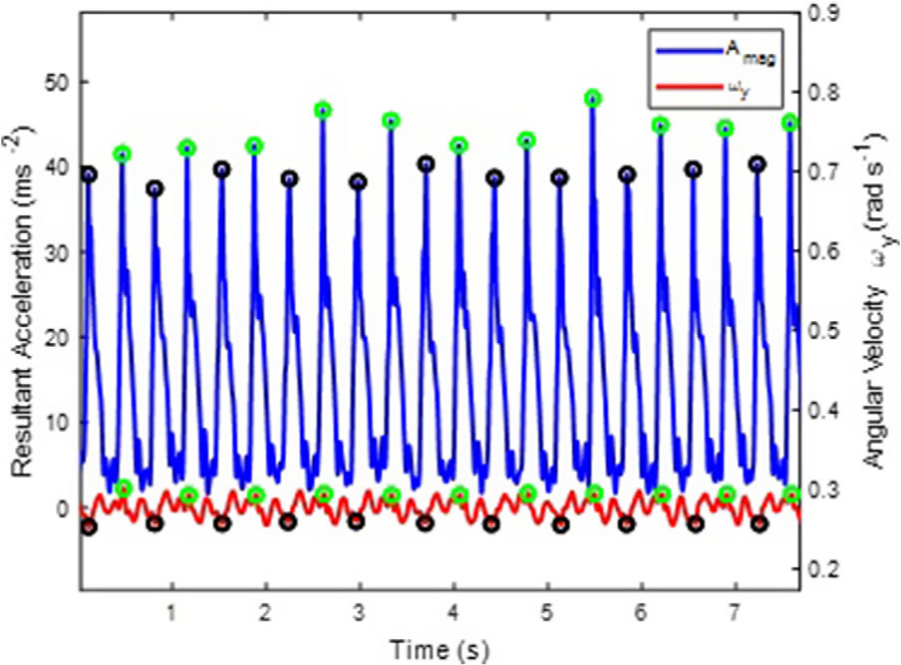
Identification of right (green circles) and left (black circles) sacral acceleration peaks (blue) based on angular velocity (red) maxima (green circles) and minima (black circles) about the anterior-posterior axis.

For each peak in A_mag_ found, the algorithm assesses the corresponding ω_y_ peak or trough. If the one found is a maximum, then the A_mag_ peak is happening during the right impact and vice versa (Figure 6).

## 3. Results and discussion

**Figure 7.**
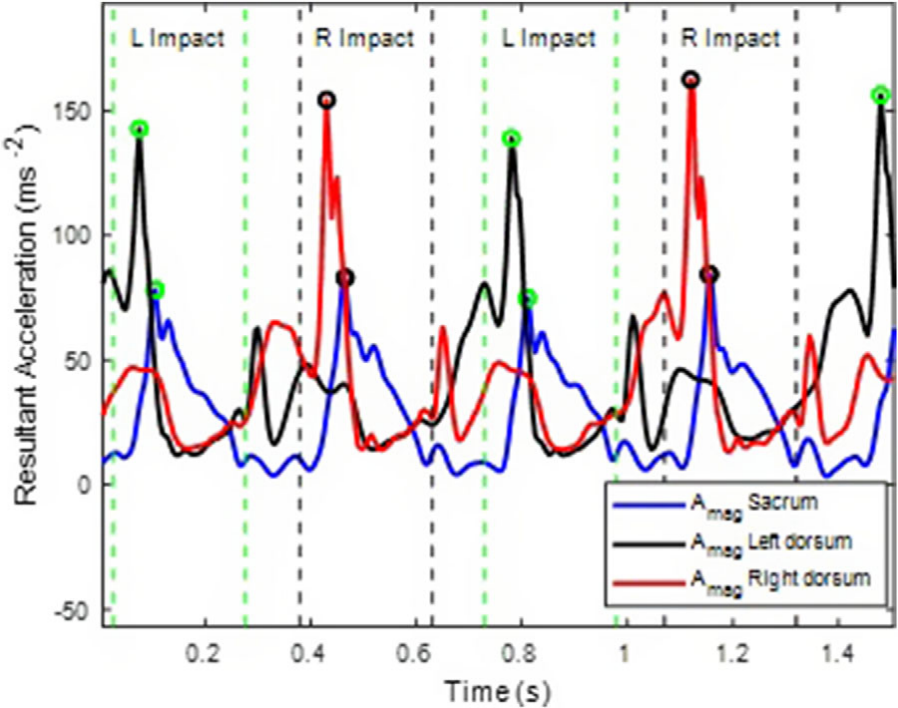
Comparison of right- and left-side impacts recorded by the sacral IMU (blue) with those from the right (red) and left (black) dorsum IMUs by Chebbi et al. (2023).

Results from the previous method (Chebbi et al., 2023) showed that during the right impact, sacral angular velocity increased, and the RIC was identified as the first peak after maximum Sacral ω_y_. Furthermore, during the left impact, sacral angular velocity decreased, and the LIC was identified as the first peak after minimum Sacral ω_y_. The mean and standard deviation of the time difference between RIC, LIC as determined from foot and sacrum data was 3.7 ± 1.7 ms in LG, 6.1 ± 2.2 ms in IN, and 2.8 ± 1.6 ms in DE for the right side and 3.8 ± 1.8 ms in LG, 5.8 ± 1.8 in IN, and 3.1 ± 1.5 ms in DE for the left side (Figure 7). Although this difference in time is expected as the wave propagation takes time to travel from the foot to the sacrum, the method was able to identify the side peaks as long as they were not missing.

The previous method (Chebbi et al., 2023) has some limitations. It assumes that the first peak identified in the acceleration data determines whether the impact is on the right or left side and then classifies all subsequent peaks based on this initial determination. This means that if the first peak is identified as a right-side impact, all alternating peaks will also be classified as right-side impacts, and vice versa for a left-side impact. This assumption may not hold true in scenarios where the runner changes direction or stride pattern, leading to alternating impacts on the right and left sides. Therefore, while this method can be effective for consistent, unidirectional running patterns, it may not accurately classify all peaks in more complex or variable running scenarios.

**Figure 8.**
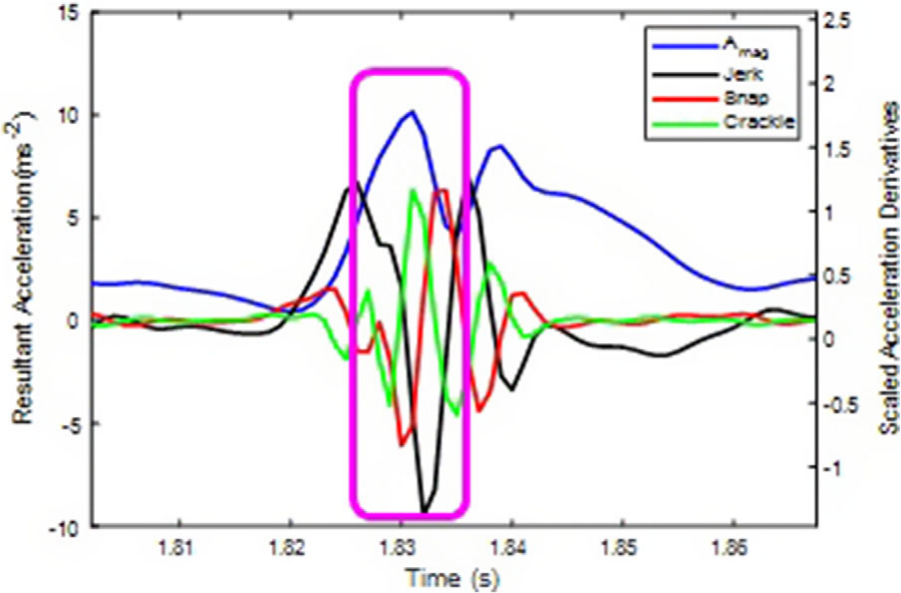
Visualization of resultant linear acceleration (blue) impact peaks highlighted by a pink square, alongside scaled Jerk (2×), Snap (3×), and Crackle (4×).

The newer method described in this work is more robust and adaptable to changes in running patterns because it treats each peak in the acceleration data independently from all others. The algorithm identifies each peak in the A_mag_ data and then uses the “crackle” function to refine the peak detection. The advantage of using “crackle” over jerk or snap lies in its sensitivity to rapid changes in acceleration, which are characteristics of the initial impact in a running cycle. While jerk and snap can provide valuable information about the overall pattern of movement (Figure 8), “crackle” offers a more nuanced view of the intricate changes occurring at each impact. This makes “crackle” particularly effective for capturing the initial peak in the acceleration data, providing a more accurate representation of the running cycle.

By analyzing each peak independently, the algorithm can adapt to changes in the running pattern, making it especially effective for outdoor running where various events can disrupt the running cycle. This peak-based approach allows for a more dynamic classification of right and left impacts, enhancing the accuracy of subsequent analyses needed for gait assessment.

**Figure 9.**
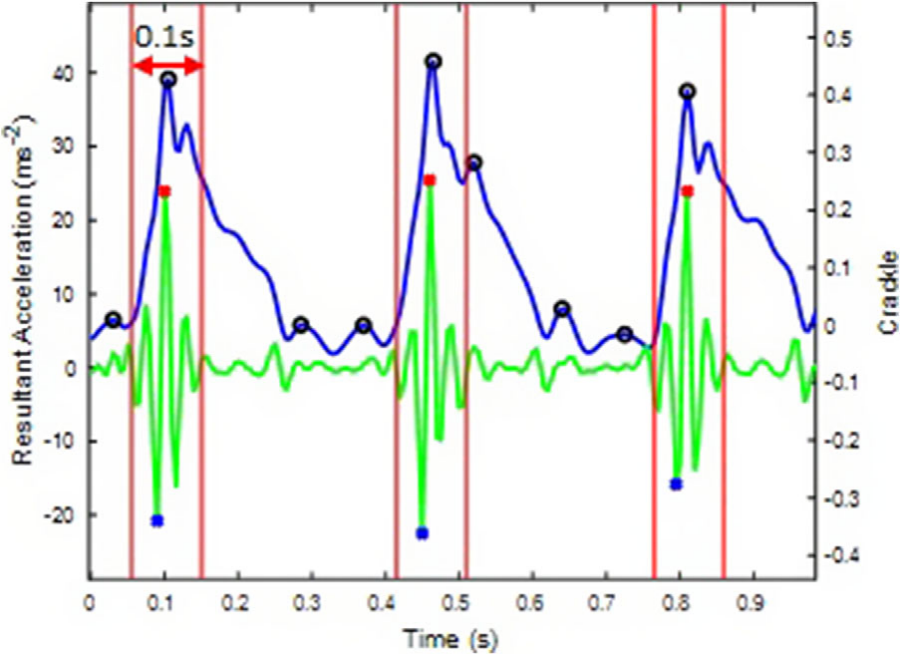
Sacral resultant acceleration (blue) impact peak (black circle) identified within a 0.1 s window around Crackle (green) peaks indicated by red stars.

In addition to the “crackle” approach (Figure 9), our study also used the ω_y_ data to validate whether the identified peak in A_mag_ corresponds to a right or left impact. This combination of methods enhances the robustness of our analysis, ensuring the accurate identification of right and left impacts, particularly crucial in complex running scenarios.

In comparison to other methods (Table 1) performed in indoor running, the new approach demonstrated high accuracy in identifying the side of foot contact during running on LG, at 99.2 ± 1.3%. This is substantially higher than the (Lee et al., 2010) method, which had an accuracy of 81.9%, and the (Benson et al., 2019) and (Auvinet et al., 2002) methods, which had accuracies of 54.6 and 75%, respectively.

**Table 1.** Accuracy (%) by method and condition (LG, IN, DE)

		Indoor running				Outdoor running	
Methods	LG	IN	DE		LG	IN	DE
Chebbi	99.2%	95.8%	99.8%		97.2%	96.5%	96.0%
Lee	81.9%	–	–		–	–	
Benson	54.6%	–	–		–	–	–
Auvinet	75%	–	–		–	–	–

The new method also performed well under more challenging conditions. Under decline conditions, the mean accuracy was 99.8 ± 0.3%. However, in incline conditions, the mean accuracy was 95.8 ± 4.7%. Despite the decrease in accuracy for graded conditions, the method still outperformed the previous methods.

For outdoor running (Table 1), the method maintained high accuracy, with an accuracy of 97.2 ± 4.8% for LG and 96.5 ± 5.3% for incline conditions. Although the accuracy decreased to 96.0 ± 5.1% under decline conditions, it still outperformed the other methods, demonstrating its robustness and reliability in various outdoor running scenarios.

To assess the effect of grade and speed on the accuracy of detecting side impacts in both indoor and outdoor environments, we conducted a two-way ANOVA. In the indoor environment, where speed was more controlled, both grade (*p* < 0.001) and speed (*p* < 0.05) had significant main effects. Their interaction (*p* < 0.001) further influenced the outcome, highlighting the combined impact of these factors on accuracy. In contrast, in the outdoor environment, only speed showed a significant main effect (*p* < 0.05).

These findings indicate that environmental conditions modulate how grade and speed influence the accuracy of detecting side impacts, with more pronounced effects and interactions observed indoors. Speed and grade affected the acceleration waveform, with different peak magnitudes in A_mag_ being impacted, which in turn affected the accuracy of detecting side impacts.

**Figure 10.**
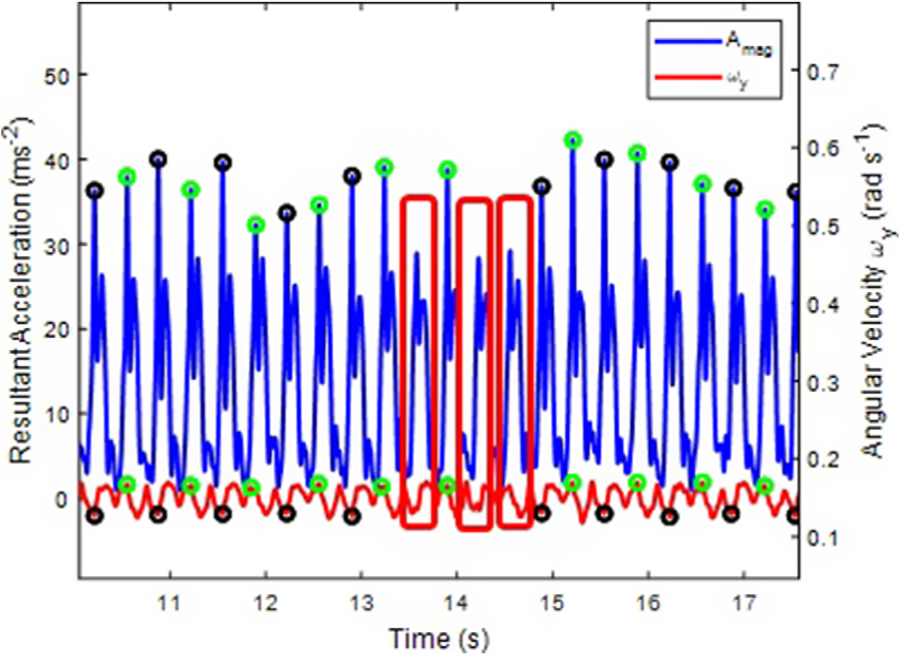
Assessment of the algorithm’s performance in identifying sacral acceleration side impact peaks. Despite missing certain peaks (marked in red), it accurately determines the side of impact for subsequent detections.

One limitation of the current method for both indoor and outdoor running is the difficulty in identifying occasional peaks in the resultant acceleration (Figure 10). This limitation arises from the nature of the peak identification function. Improving the estimation of input parameters, such as window size and peak height, for the automatic detection process across all grades and speed ranges could enhance peak detection. Despite this issue, the method remains effective. The model is resilient, accurately handling the impacts between missed peaks and after missed peaks, ensuring reliable performance in determining the side impact of subsequent detected peaks.

Another limitation arising from the outdoor running condition is that the data are concatenated in discontinued segments for the same speeds and grades over a total of 5 miles. These segments have gaps in between them, which can cause the algorithm to misidentify peaks at the edges of each of them, especially when the data corresponds to less than a complete footfall.

## 4. Conclusion

This study validates the use of a single inertial sensor placed on the sacrum as an effective tool for detecting right and left impacts during running. Building upon previous research by Wada et al. (2020) and Chebbi et al. (2023), the novel algorithm developed here leverages the “crackle” function, the third derivative of linear acceleration, to capture the peak in acceleration data associated with foot impact at IC. This method also incorporates angular velocity about the anterior-posterior axis (ω_y_) to determine whether the identified peak corresponds to a right or left foot impact.

In indoor running scenarios, this method demonstrated excellent accuracy in identifying the side of foot contact, achieving a mean accuracy of 99.2 ± 1.3% on LG, which is substantially higher than other methods. It also performed well in more challenging conditions, with accuracies of 95.8 ± 4.7% in incline conditions and 99.8 ± 0.3% in decline conditions. For outdoor running, the method was also determined to be highly accurate, with a mean accuracy of 97.2 ± 4.8% on LG, 96.5 ± 5.3% under incline conditions, and 96.0 ± 5.1% under decline conditions. These results highlight the method’s robustness and reliability across various running scenarios, both indoor and outdoor, and demonstrate that this method outperformed other existing methods.

The two-way ANOVA results indicate that environmental conditions modulate how grade and speed influence the accuracy of detecting side impacts, with more pronounced effects and interactions observed indoors. Speed and grade affected the acceleration waveform, with different peak magnitudes in A_mag_ being impacted, which in turn affected the accuracy of detecting side impacts.

Future work will focus on further improving the method’s robustness and reliability in real-world scenarios. Enhancing the sacral IMU’s capabilities to accurately identify GEs, including IC and toe-off events, will be a key objective. By applying this comprehensive method, we can effectively track key running metrics in both indoor and outdoor environments.

## Data Availability

Data sharing is not applicable to this article as no new data were created or analyzed in this study.
